# Surviving the Nightmare: Massive Bleeding From Large Intraoral Arteriovenous Malformation During Airway Management for Angioembolization Procedure

**DOI:** 10.1155/cria/6311200

**Published:** 2024-12-19

**Authors:** Muhammad Faisal Khan, Muhammad Khuzzaim Khan, Sidra Nazir, Faisal Shamim

**Affiliations:** ^1^Department of Anaesthesiology, Aga Khan University Hospital, Karachi, Pakistan; ^2^Department of Internal Medicine, Dow University of Health Sciences, Karachi, Pakistan

**Keywords:** angioembolization, aretriovenous malformation, difficult airway, fiberoptic intubation, video laryngoscopy

## Abstract

Arteriovenous malformations (AVMs) in the head and neck present significant challenges due to airway management complexities and hemorrhage risks. This case report describes a 15-year-old female with a congenital facial AVM causing dyspnea and obstructive symptoms. The patient required angioembolization of the AVM, but many hospitals deferred the procedure due to the anticipated difficult airway and severe bleeding risks. We did two attempts of awake fiberoptic intubation but could not succeed due to patient intolerance. Subsequently, inhalational induction started and video laryngoscopy performed but also failed due to anatomical distortion. With attempts to bag mask ventilate, severe venous engorgement started and patient experienced massive hemorrhage and circulatory collapse, necessitating prolonged resuscitation and intubation efforts. Eventually, intubation was successful after 40 min using suction assisted laryngoscopy and decontamination (SALAD) technique by video laryngoscope. She underwent angioembolization and shifted to the ICU where she remained on mechanical ventilation for 9 days. After tracheostomy was performed, she was gradually weaned off from ventilator and was later discharged. This case highlights the need for meticulous planning, comprehensive airway evaluation, backup strategies, and multidisciplinary support, suggesting video laryngoscopy as a valuable alternative in high-bleeding-risk cases.

## 1. Introduction

Arteriovenous malformations (AVMs) are congenital vascular anomalies that can expand over a person's lifetime. They frequently occur in the head and neck region and can lead to significant clinical issues such as pain, swelling, bleeding, and infection [1]. These lesions create abnormal connections between arteries and veins, lacking the usual intervening capillary bed, forming tangled vessel networks known as niduses [2]. They can be associated with craniofacial arteriovenous metameric syndrome, affecting specific intracranial and facial regions. AVMs can cause hemorrhage, cosmetic, and psychosocial morbidity. Intraoral AVMs have unique challenges due to their propensity to cause significant airway obstruction and bleeding [3]. These are further compounded during surgical or interventional procedures, where airway management becomes critically difficult [4]. The infiltrative nature of intraoral AVMs can distort the anatomy, complicating the visualization and access required for intubation [5]. Guidelines for managing difficult airways, such as those provided by the American Society of Anaesthesiologists, emphasize the importance of having a detailed preoperative plan, availability of advanced airway devices, and the readiness to perform surgical airway techniques when necessary [6].

This case is significant because it highlights the complex challenges of managing a pediatric patient with a large oropharyngeal AVM, a condition that was deferred by multiple hospitals due to its high risk. The case demonstrates the critical need for meticulous planning, advanced airway management techniques, and a multidisciplinary approach to ensure a successful outcome.

## 2. Case Presentation

**Figure 1:**
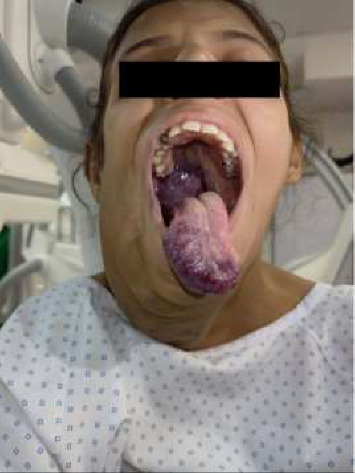
Clinical presentation of the patient showing the compressible lesion extending from the right cheek to the neck up to the thoracic inlet and crossing the midline.

A 15-year-old female with a congenital facial AVM that had been progressively increasing in size presented for angioembolization. She experienced dyspnea when lying flat and dysphagia for solids but could tolerate liquids and semisolids and had a nasal voice quality. Examination revealed a compressible lesion extending from the right cheek to the neck up to the thoracic inlet, crossing the midline (Figure 1). Her mouth opening was more than three fingers, with a Mallampati score of class I, bucked teeth, and involvement of the tongue and posterior pharyngeal wall.

**Figure 2:**
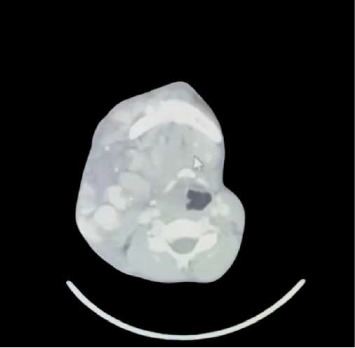
CT scan images revealing the extent of the vascular malformation. The malformation extends from the infratemporal fossa to the thoracic inlet, partially obstructing the oropharynx and nasopharynx, displacing the larynx to the left, and narrowing it slightly.

Preoperative workup showed a hemoglobin level of 9.6 g/dL. A prior CT scan showed vascular malformation extending from the infratemporal fossa to the thoracic inlet, partially obstructing the oropharynx and nasopharynx, displacing the larynx to the left, and narrowing it slightly (Figure 2). The lesion also extended deep into the soft tissue of the tongue. The patient had been denied the procedure at multiple hospitals due to the risk of bleeding and inoperable nature of lesion.

The patient and her family were counseled about the anticipated difficult airway and risk of massive bleeding. Blood products and an ICU bed were arranged preoperatively. A second consultant anesthesiologist was taken on board in anticipation of the challenging airway. Difficult airway cart was mobilized from the main operating room (OR) to the interventional radiology suite. Preoperative airway ultrasound in the procedure room was performed to localize the cricothyroid membrane, but it was challenging due to airway deviation by the lesion.

The initial plan was awake fiberoptic intubation, which was attempted twice but abandoned due to patient noncooperation and obstruction in the left nostril. The backup plan involved inhalational induction and check laryngoscopy. After successful bag-mask ventilation, the first attempt with a CMAC video laryngoscope failed to pass beyond the base of the tongue. The patient then began to obstruct due to venous engorgement, leading to tongue and mass swelling, and started bleeding from the oral cavity, making ventilation difficult. The patient desaturated rapidly, and massive bleeding ensued. Multiple laryngoscopy attempts were unsuccessful, leading to bradycardia and pulseless electrical activity (PEA). CPR was initiated, a code blue was called, and a massive transfusion protocol (MTP) was activated. The pediatric code blue team assisted in resuscitation efforts.

**Figure 3:**
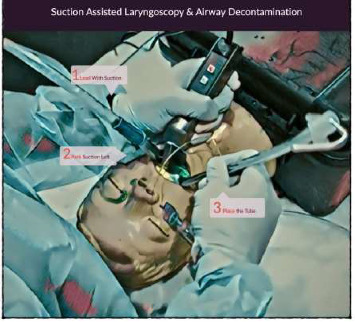
Suction-assisted laryngoscopy and airway decontamination (SALAD) technique.

Front-of-neck airway access, such as cricothyroidotomy or tracheostomy, was considered but precluded due to the midline extension of the AVM. After 40 min of continuous efforts, the patient was successfully intubated using the suction-associated laryngoscopy and decontamination (SALAD) technique through video laryngoscopy and the cuffed endotracheal tube (ETT) of size 5.5 was inserted. Immediately, patient's spO2 improved and once the patient's blood pressures stabilized on norepinephrine infusion, angioembolization proceeded to control persistent oral bleeding (Figure 3). The oral and nasal cavities were packed with transamine and adrenaline-soaked packs. The patient was resuscitated with 4 units of packed red blood cells (PCV), 4 units of fresh frozen plasma (FFP), 6 units of platelets, 500 mL of colloid, and 2.5 L of crystalloids. Invasive monitoring was placed, and the patient was shifted to the pediatric intensive care unit (PICU) postprocedure. The next day, an off-sedation trial was given, and her Glasgow Coma (GCS) score was E4M5Vt (10/15). Inotropes and vasopressors were gradually reduced and stopped.

On the third day, an ENT attempt to remove the packing at the bedside resulted in bleeding from the oral cavity. On the fourth day, the patient was taken to the OR for sclerotherapy and a feeding gastrostomy. By the ninth day, ENT was persuaded to perform a tracheostomy, which was done with the help of radiology marking the site via ultrasound. The patient was then weaned off the ventilator and subsequently stepped down to special care and then to the ward. She was discharged on the 23rd day. The patient was followed up monthly in the plastic surgery clinic, where she received sclerotherapy injections to the lesion twice. After 6 months, she tolerated a capping trial for the tracheostomy. The patient is now being managed as a palliative care case.

## 3. Discussion

This case report describes the significant challenges encountered during airway management in a patient with an extensive AVM undergoing angioembolization in a radiology suite.

AVMs, like the one presented here, can pose significant challenges for airway management due to several factors [7]. The abnormal connections between arteries and veins can lead to high-flow shunting, potentially complicating bag-mask ventilation. In addition, the infiltrative nature of AVMs can distort airway anatomy, hindering visualization of the glottis during laryngoscopy and increasing the risk of bleeding during airway manipulation [8].

A thorough preoperative assessment is crucial in patients with AVMs, including evaluation of airway patency and a history of respiratory infections, which can exacerbate airway edema [9]. In this case, the patient's history of chest infection and the extensive nature of the AVM limited the available airway management options. Awake fiberoptic intubation, the gold standard and preferred technique for many anticipated difficult airways, was not feasible due to patient uncooperation. In a recent case report by Ghosh et al., the authors described a successful technique of securing airway in a pregnant patient with large intraoral AVM. They used a pediatric fiberoptic bronchoscope and inserted a thin calibrated guidewire through the suction channel. The scope along with wire was passed into trachea, pushed guidewire to a considerable length, removed the scope, and then passed airway exchanged catheter (AEC) over the guide wire. Subsequently, a wire spiral 6.0 mm tracheal tube was slowly railroaded over the AEC [10].

Inhalational induction with spontaneous breathing is the next safe approach in the management of anticipated difficult airway [11, 12]. We opted this method and were initially able to perform laryngoscopy, but distorted anatomy leads our first attempt unsuccessful.

Successful intubation was achieved with constant suction and video laryngoscope. SALAD is the term coined by Jim Du Canto and colleagues for a suction method used to preventing airway soiling during laryngoscopy when there is profuse regurgitation [13]. The technique involves using a large-bore suction catheter in conjunction with direct laryngoscopy to clear the airway of fluids, blood, or other debris. This is particularly useful in cases where traditional laryngoscopy might be hindered by poor visibility due to secretions or other obstructions. Large-scale, randomised controlled trials comparing SALAD with traditional emergency airway care techniques in vivo are currently lacking. The reason would be the lack of designing and conducting research comparing SALAD with conventional procedures due to the unexpected nature of large airway contamination during an intubation attempt. But as the method has become more widely known, there is a growing body of evidence evaluating the technique in simulation and assessing its benefit to learners.

Our patient's successful outcome, with eventual decannulation and removal of the gastrostomy tube, underscores the importance of a multidisciplinary approach and perseverance in managing these challenging cases. Notably, the patient did not experience any complications related to hypoxic brain injury or blood aspiration, highlighting the effectiveness of the implemented strategies despite the initial difficulties.

In conclusion, airway management in patients with extensive AVMs undergoing procedures outside of the OR presents significant challenges. A thorough preoperative assessment, a well-equipped difficult airway cart, and effective collaboration between anesthesia, ENT, and radiology teams are crucial for successful management.

## Data Availability

The data that support the findings of this study are available from the corresponding author upon reasonable request.
